# Formation of extended polyiodides at large cation templates

**DOI:** 10.1107/S2053229624004194

**Published:** 2024-05-13

**Authors:** Alexander J. Blake, Carlo Castellano, Vito Lippolis, Enrico Podda, Martin Schröder

**Affiliations:** a School of Chemistry, University of Nottingham, University Park, Nottingham, NG7 2RD, United Kingdom; b Dipartimento di Chimica, Università degli Studi di Milano, Via Golgi 19, Milano, 20133, Italy; c Dipartimento di Scienze Chimiche e Geologiche, Università degli Studi di Cagliari, S.S. 554 Bivio per Sestu, Monserrato (CA), 09042, Italy; d Centre for Research University Services (CeSAR), Università degli Studi di Cagliari, S.S. 554 Bivio per Sestu, Monserrato (CA), 09042, Italy; e Department of Chemistry The University of Manchester Manchester M139PL United Kingdom; University of Strathclyde, United Kingdom

**Keywords:** crystal structure, mixed-donor macrocycles, [2.2.2]cryptand, palladium(II) complexes, diiodine, polyiodides, FT-Raman spectroscopy

## Abstract

By studying the structures of (μ-1,4,10,13-tetra­thia-7,16-di­aza­cyclo­octa­deca­ne)bis[iodidopalladium(II)] diiodide penta­(diiodine), [Pd_2_I_2_(C_12_H_26_N_2_S_4_)](I)_2_·5I_2_ or [Pd_2_I_2_([18]aneN_2_S_4_)](I)_2_·(I_2_)_5_, and 4,7,13,16,21,24-hexa­oxa-1,10-diazo­niabi­cyclo­[8.8.8]hexa­cosane triiodide iodide hemi­penta­(diiodine) di­chloro­methane mono­solvate, C_18_H_38_N_2_O_6_^2+^·I_3_^−^·I^−^·2.5I_2_·CH_2_Cl_2_ or [H_2_([2.2.2]cryptand)](I_3_)(I)(I_2_)_2.5_·CH_2_Cl_2_, we confirm the structural variety of extended polyiodides achievable upon changing the shape, charge and dimensions of the cation template, by altering the synthetic strategy adopted and/or the experimental conditions. Although it is still often difficult to characterize discrete [I_2*m*+*n*_]^*n*−^ polyiodides higher than I_3_^−^ on the basis of structural parameters, such as I—I bond distances, FT–Raman spectroscopy appears to identify them as aggregates of I_2_, I^−^ and (symmetric or slightly asymmetric) I_3_^−^ building blocks linked by I⋯I inter­actions of varying strengths. However, because FT–Raman spectroscopy carries no information about the topological features of extended polyiodides, the two techniques should therefore be applied in combination to enhance the analysis of this kind of compounds.

## Abstract 2

Analysis of the structures of [Pd_2_I_2_([18]aneN_2_S_4_)](I)_2_·(I_2_)_5_ and [H_2_([2.2.2]cryptand)](I_3_)(I)(I_2_)_2.5_·CH_2_Cl_2_ identify some of the factors responsible for the structural features of extended polyiodides.

## Introduction

Among extended anionic inorganic frameworks, the formation of polyhalides (Sonnenberg *et al.*, 2020 ▸; Aragoni *et al.*, 2003 ▸, 2022 ▸) and, in particular, polyiodides represents a remarkable example of supra­molecular self-assembly (Blake *et al.*, 1998*c* ▸; Svensson *et al.*, 2003 ▸), and it continues to capture the inter­est of many researchers in the field (Savastano, 2021 ▸; Savastano *et al.*, 2022 ▸; Horn *et al.*, 2003*a* ▸,*b* ▸; Aragoni *et al.*, 2004 ▸, 2023*a* ▸) due to the richness of its unpredictable and puzzling structural chemistry, and inter­esting applicative possibilities (Paulsson *et al.*, 2004 ▸; Yin *et al.*, 2012 ▸; Fei *et al.*, 2015 ▸). Iodine and iodides together tend to catenate (Arca *et al.*, 1999 ▸; Garau *et al.*, 2022 ▸) *via* the combination of (Lewis acidic) I_2_ with (Lewis basic) I^−^/I_3_^−^ building blocks (Ciancaleoni *et al.*, 2016 ▸). This affords extended arrays exhibiting a range of topologies, and these are highly dependent on the size, shape and charge of the countercation acting as a template. Some polyiodides are present in the crystal structure as discrete aggregates, but frequently they form extended networks in which the identification of the basic repeat unit of general formula [I_*n*_(I_2_)_*m*_]^*n*−^ or [I_2*m*+*n*_]^*n*−^ (*n*, *m* > 0) can become arbitrary. Consequently, they are better described as aggregates of I_2_, I^−^ and I_3_^−^, held together by I⋯I inter­actions of varying strengths, from rather strong (*ca* 3.3 Å) to fairly weak, up to the van der Waals contact distance (*ca* 4 Å). Our inter­est in this field has been mainly focused on the use of metal complexes of macrocyclic ligands (mainly thio­ether crowns) as templating cations for controlling the self-assembly of extended polyiodide arrays (Blake *et al.*, 1996 ▸, 1998*a* ▸,*b* ▸). These complex cations are relatively chemically inert and their shape, size and charge can be changed readily, thus providing cationic templates for different targeted polyiodide topologies. Furthermore, we have also been inter­ested in the reactivity of macrocyclic ligands with I_2_ and inter-halogens I*X* (*X* = Br and Cl) to better understand the structural nature of the resulting products (Blake *et al.*, 1997 ▸). The formation of polyiodide networks featuring spirals, belts, ribbons, sheets and cages as their structural motifs has been achieved either by reacting the PF_6_^−^ or BF_4_^−^ salts of the complex cation 
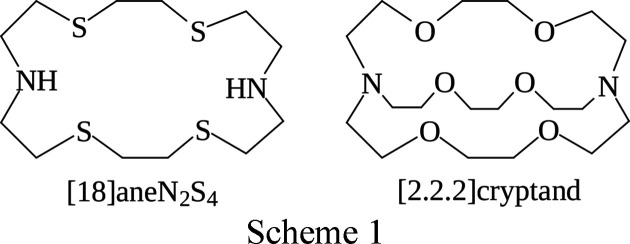
templates with an excess of I_2_ in a single phase, or by addition of an NaI/I_2_ mixture in a single phase, the preferred polyiodide being formed *via* self-assembly. As a further example of the versatility of this synthetic approach to the formation of multidimensional polyiodide networks, we report here the use of the metal complex [Pd_2_Cl_2_([18]aneN_2_S_4_)](PF_6_)_2_ ([18]aneN_2_S_4_is 1,4,10,13-tetra­thia-7,16-di­aza­cyclo­octa­decane; see Scheme 1) and the neutral [2.2.2]cryptand (4,7,13,16,21,24-hexa­oxa-1,10-di­aza­bicyclo­[8.8.8]hexa­cosa­ne) (Scheme 1) as templates in the reaction with I_2_.

## Experimental

### Materials and methods

All starting materials, including [18]aneN_2_S_4_ and [2.2.2]cryptand, and solvents, were obtained from Aldrich or Merck and were used without further purification. [Pd_2_Cl_2_([18]aneN_2_S_4_)](PF_6_)_2_ was prepared according to the literature (Blake *et al.*, 1990 ▸). Microanalytical data were obtained on a Fisons EA 1108 CHNS-O instrument operating at 1000 °C. FT–Raman spectra (resolution 4 cm^−1^) were recorded on a Bruker RF100FTR spectrometer fitted with an indium–gal­lium–arsenide detector operating at room temperature with an excitation wavelength of 1064 nm (Nd:YAG laser). No sample decomposition was observed during the experiments at the power level of the laser source used between 20 and 40 mW. The values in parentheses next to the values represent the intensities of the peaks relative to the strongest, which is taken to be equal to 10.

### Synthesis and crystallization

#### Synthesis of (I)

To a solution of [Pd_2_Cl_2_([18]aneN_2_S_4_)](PF_6_)_2_ (17.1 mg, 0.019 mmol) in MeCN (4 ml) was added a solution of I_2_ (17.7 mg, 0.070 mmol) in MeCN (4 ml). No precipitate formed upon mixing, but dark-brown prismatic crystals of title compound (I) (Scheme 2) formed after several days by slow evaporation of the solvent from the reaction mixture. These were isolated from the mother liquor and washed with diethyl ether (8.4 mg, 36.3% yield). Elemental analysis found [calculated (%) for C_6_H_13_I_7_NPdS_2_]: C 6.28 (6.22), H 1.15 (1.13), N 1.24 (1.21), S 5.52 (5.54). FT–Raman (range 500–50 cm^−1^): ν(I–I) 169.7 (10).
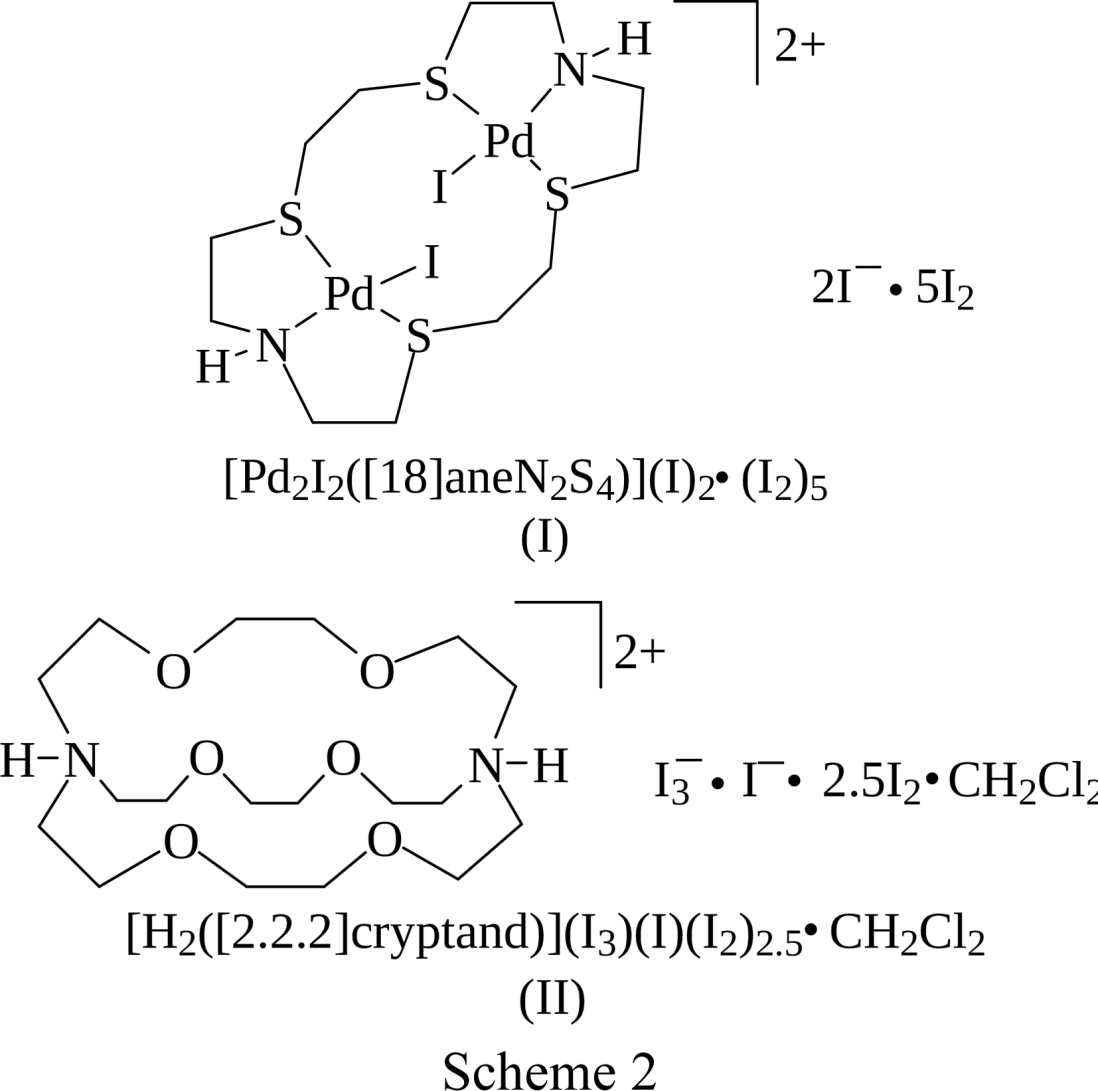


#### Synthesis of (II)

To a solution of [2.2.2]cryptand (20 mg, 0.053 mmol) in CH_2_Cl_2_ (4 ml) was added a solution of I_2_ (53.8 mg, 0.212 mmol) in CH_2_Cl_2_ (4 ml). A dark-brown microcrystalline precipitate corresponding to the formulation of title compound (II) (Scheme 2) formed immediately. This was isolated by filtration and washed with diethyl ether (58.5 mg, 77.3% yield). Elemental analysis found [calculated (%) for C_19_H_40_Cl_2_I_9_N_2_O_6_]: C 14.25 (14.21), H 2.47 (2.51), N 1.80 (1.75). FT–Raman (range 500–50 cm^−1^): ν(I–I) 167.40 (10), 149.8 (6), 106.1 (5). Dark-brown prismatic single crystals suitable for X-ray diffraction analysis were grown from a solution of the obtained solid in MeCN by slow evaporation of the solvent.

### Refinement of X-ray crystal structures

Crystal data, data collection and structure refinement details are summarized in Table 1 ▸. H atoms were placed geometrically and refined isotropically riding on their parent C atoms, with *U*_iso_(H) = 1.2*U*_eq_(C). For (II), H atoms bonded to quaternary N atoms could be located from the difference Fourier map and their positions were refined freely. *OLEX2* (Dolomanov *et al.*, 2009 ▸) was used both as the graphical inter­face for the structural investigation and for the preparation of the figures.

## Results and discussion

### Synthesis and crystal structures

**Figure 1:**
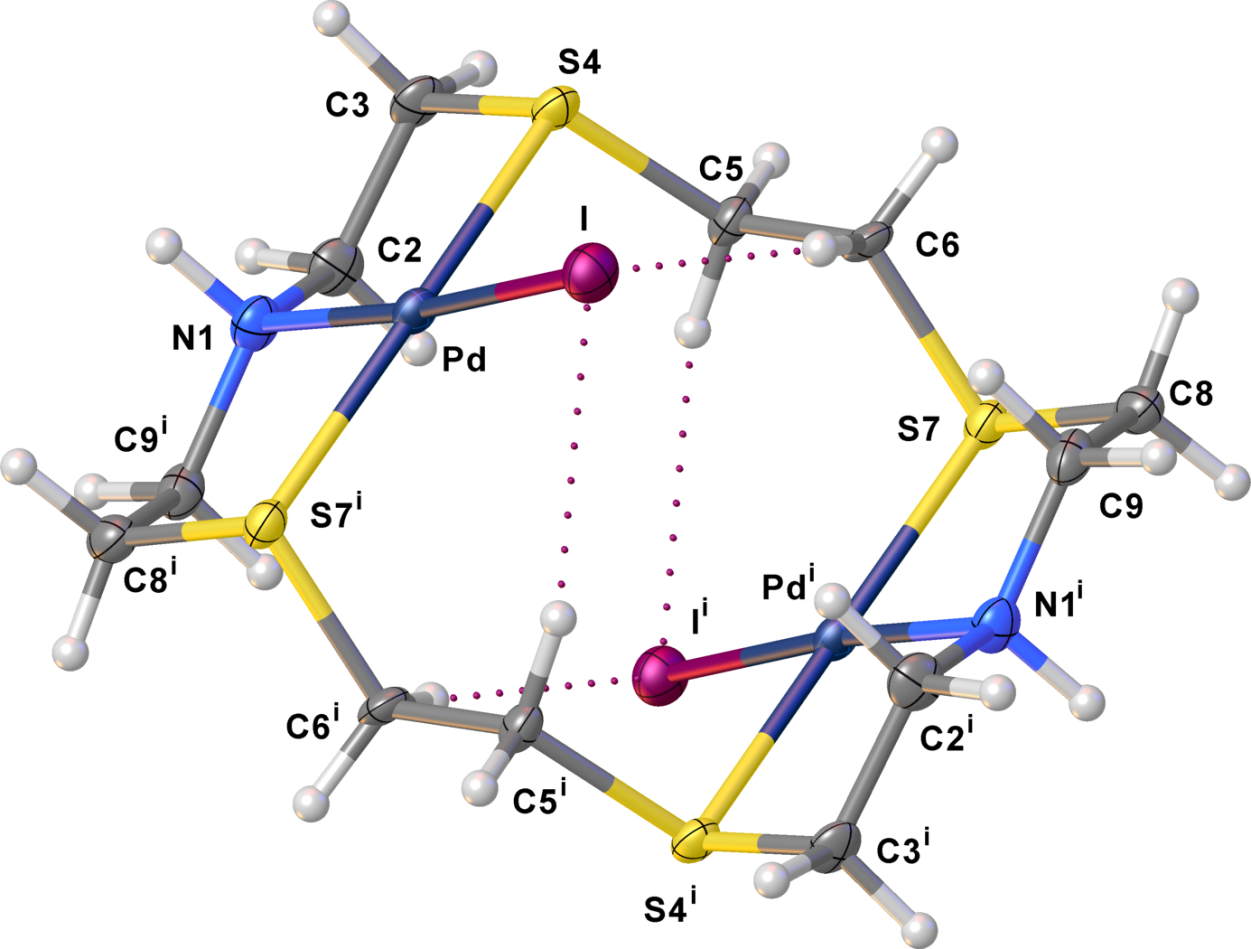
View of the dication in (I), showing the atom-numbering scheme adopted. Displacement ellipsoids are drawn at the 50% probability level. Intra­molecular C—H⋯I hydrogen bonds: C6⋯I = 3.779 (11), H6*A*⋯I = 2.84, C5^i^⋯I = 3.755 (10), H5*A*⋯I = 2.80 Å, C6—H6*A*⋯I = 162 and C5—H5*A*⋯I^i^ = 161°. [Symmetry code: (i) −*x* + 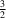, −*y* + 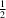, −*z* + 1.]

Previously, we have reported the crystal structures of [Pd_2_Cl_2_([18]aneN_2_S_4_)](I_3_)_2_ (Blake *at al.*, 1998*c* ▸,*d* ▸) and [Pd_2_Cl_2_([18]aneN_2_S_4_)]_1.5_(I_5_)(I_3_)_2_ (Blake *et al.*, 1998*a* ▸,*c* ▸) obtained from the reaction in MeCN of [Pd_2_Cl_2_([18]aneN_2_S_4_)](PF_6_)_2_ with ^*n*^Bu_4_NI and I_2_ in 1:2:2 and 1:2:4 molar ratios, respectively. In the former compound, dinuclear palladium(II) complexes are linked *via* Pd⋯I con­tacts into ⋯I_3_^−^⋯I_3_^−^⋯I_3_^−^⋯ sinusoidal chains. In [Pd_2_Cl_2_([18]aneN_2_S_4_)]_1.5_(I_5_)(I_3_)_2_, [Pd_2_Cl_2_([18]aneN_2_S_4_)]^2+^ cat­ions are held together by N—H⋯Cl hydrogen bonds and occupy channels formed within the self-assembled three-dimensional (3D) polyiodide network. This network is made up of offset layers of stacked poly-I_3_^−^ moieties (in­cluding those I_3_^−^ belonging to the I_5_^−^ units), featuring fused 14- and 24-membered rings inter­woven by I_8_^2−^ units (I_5_^−^⋯I_3_^−^). We sought to attempt also the direct reaction of [Pd_2_Cl_2_([18]aneN_2_S_4_)](PF_6_)_2_ with I_2_ in the absence of pre­formed I^−^ (see §2.2.1) and, surprisingly, this afforded a dif­ferent compound corresponding to the formulation [Pd_2_I_2_([18]aneN_2_S_4_)]I_12_, as deep-red column-shaped crystals. A single-crystal X-ray structure determination (see Table 1 ▸ for crystal data) showed an asymmetric unit consisting of half a [Pd_2_I_2_([18]aneN_2_S_4_)]^2+^ dication, one iodide anion inter­acting with two crystallographically-independent I_2_ mol­ecules, and an additional half-occupied I_2_ mol­ecule disordered across a twofold axis parallel to the *b* axis. One of the two I atoms is located on a glide plane, thus defining an overall stoichiometry of [Pd_2_I_2_([18]aneN_2_S_4_)](I)_2_·(I_2_)_5_, (I), for the obtained salt. The complete dication is generated through an inversion centre and features the hexa­dentate macrocycle binding to the two metal centres *via* NS_2_ coordination. A distorted square-planar coordination geometry at each Pd^II^ metal ion is completed by coordinated iodide anions that have replaced the chloride ions (Fig. 1 ▸) in the starting material upon reaction with I_2_ (see Table 2 ▸ for selected geometric parameters).

**Figure 2:**
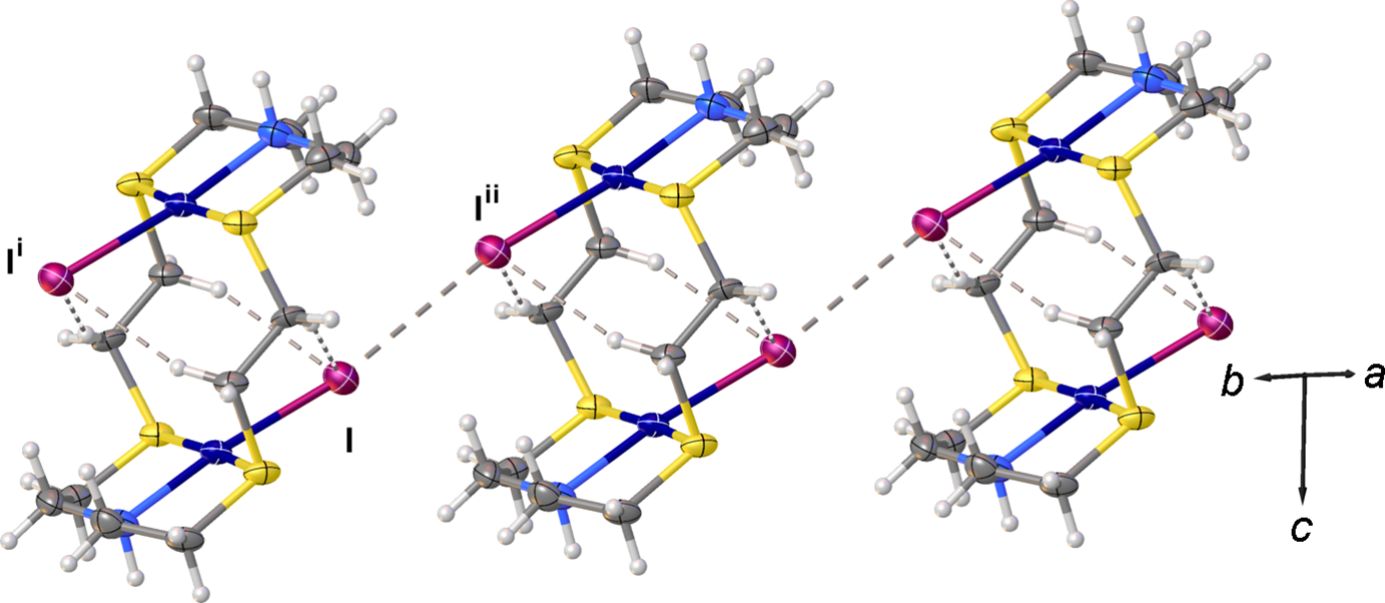
View of a chain of inter­acting [Pd_2_I_2_([18]aneN_2_S_4_)]^2+^ dications found in the crystal structure of (I). The dications are arranged into chains *via* I⋯I inter­actions of 3.545 (2) Å running along the *b* axis. [Symmetry codes: (i) −*x* + 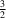, −*y* + 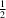, −*z* + 1; (ii) −*x* + 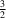, −*y* − 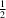, −*z* + 1.]

In (I), the Pd—N [Pd—N1 = 2.076 (9) Å] and Pd—S [Pd—S4 = 2.314 (3) and Pd—S7^i^ = 2.313 (3) Å; symmetry code: (i) −*x* + 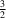, −*y* + 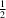, −*z* + 1] distances are very close to those observed in previously reported [Pd_2_Cl_2_([18]aneN_2_S_4_)]^2+^ dications (Blake *et al.*, 1990 ▸, 1998*a* ▸,*c* ▸,*d* ▸), while the Pd—I bond distance [2.5722 (14) Å] is significantly longer than the Pd—Cl distances [2.305 (4)–2.374 (1) Å]. As with the [Pd_2_Cl_2_([18]aneN_2_S_4_)]^2+^ dications reported previously, the dications in (I) adopt a stepped conf­ormation. Inter­estingly, in [Pd_2_Cl_2_([18]aneN_2_S_4_)]_1.5_(I_5_)(I_3_)_2_(Blake *et al.*, 1998*a* ▸,*c* ▸), the dications are linked pairwise by hydrogen bonds between the (N)H and Cl atoms to form extended chains. The [Pd_2_I_2_([18]aneN_2_S_4_)]^2+^ dications in (I) are linked by inter­molecular I⋯I contacts of 3.545 (2) Å to form chains running parallel to the *b* axis (Fig. 2 ▸). In both compounds, the complex dications feature inter­molecular inter­actions of the type C—H⋯*X* (*X* = Cl and I) (see Figs. 1 ▸ and 2 ▸).

**Figure 3:**
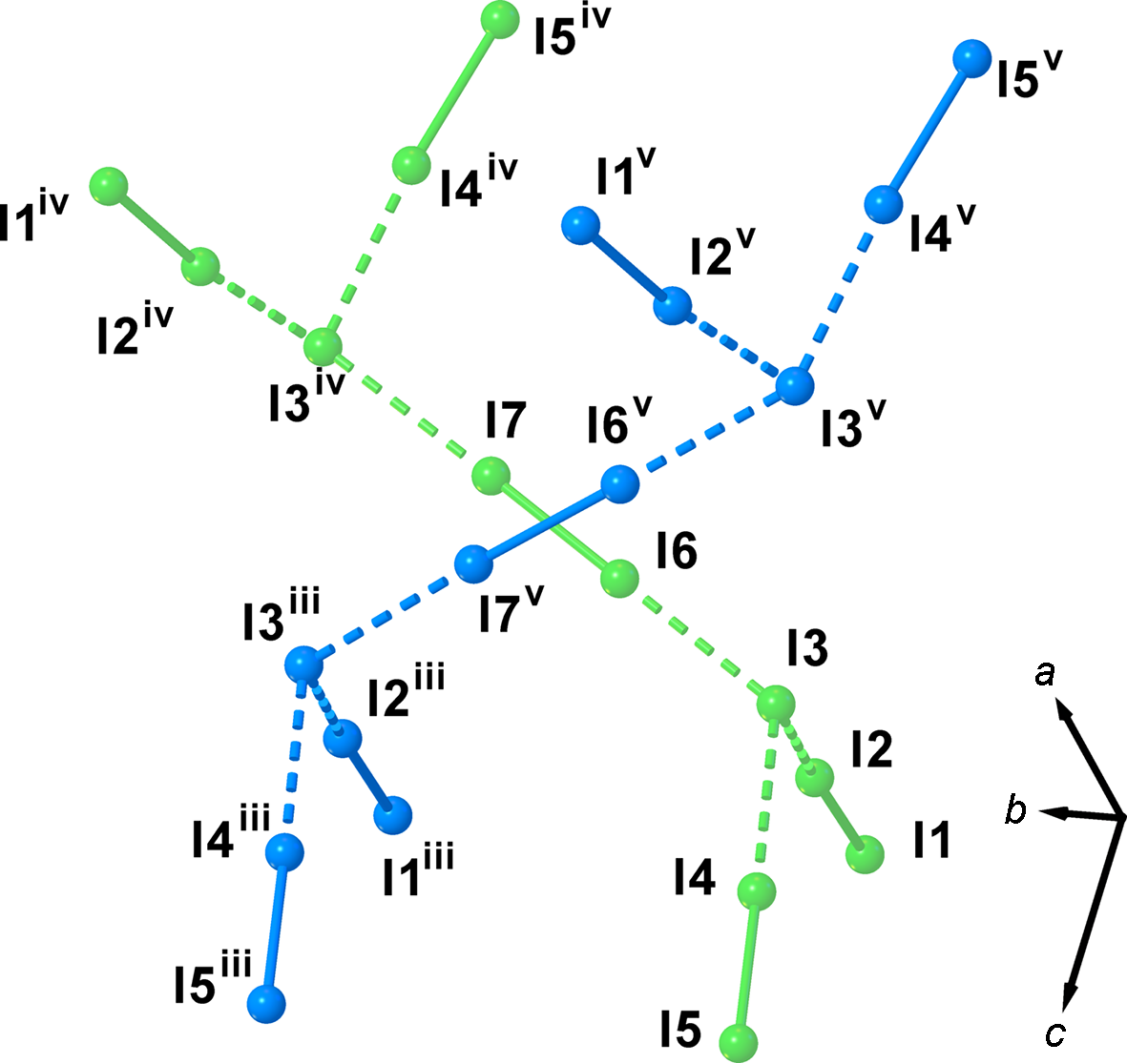
View of the two symmetry-related I_12_^2−^ anions in (I) formed by the inter­action of the two components of the disordered I_2_ mol­ecules with two ‘V-shaped’ I_5_^−^ moieties of the type [(I^−^)·(I_2_)_2_]. [Symmetry codes: (iii) *x*, *y* + 1, *z*; (iv) −*x* + 1, *y* + 1, −*z* + 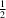; (v) −*x* + 1, *y*, −*z* + 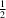.]

The polyiodide network in (I) can also be regarded as comprising I_12_^2−^ anions (Fig. 3 ▸) built up by [(I^−^)_2_·(I_2_)_5_] adducts formed by inter­action of the disordered I_2_ mol­ecules (I6—I7) [2.771 (4) Å] and ‘V-shaped’ I_5_^−^ of the type [(I^−^)·(I_2_)_2_] with the iodide anion (I3) inter­acting with two crystallographically-independent I_2_ mol­ecules [I1—I2 and I4—I5: I1—I2 = 2.7899 (15), I2⋯I3 = 3.1214 (16), I4—I5 = 2.7644 (16), I3⋯I4 = 3.205 (2) Å, I1—I2⋯I3 = 173.23 (5), I3⋯I4—I5 = 173.60 (5) and I2⋯I3⋯I4 = 95.36 (4)°].

Each component of the disordered and half-occupied I_2_ mol­ecule inter­acts at both I atoms with the iodide atom (I3) of the I_5_^−^ moiety *via* I⋯I inter­actions of 3.351 (3) (I3⋯I6) and 3.432 (3) Å [I7⋯I3^iv^; symmetry code: (iv) −*x* + 1, *y* + 1, −*z* + 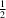]. This gives rise to two I_12_^2−^ anions in the structure, which are symmetry-related by a screw axis parallel to the *b* axis and a glide plane (the same symmetry elements that relate the two disorder components of the half-occupied I6—I7 diiodine mol­ecule) (Fig. 3 ▸).

**Figure 4:**
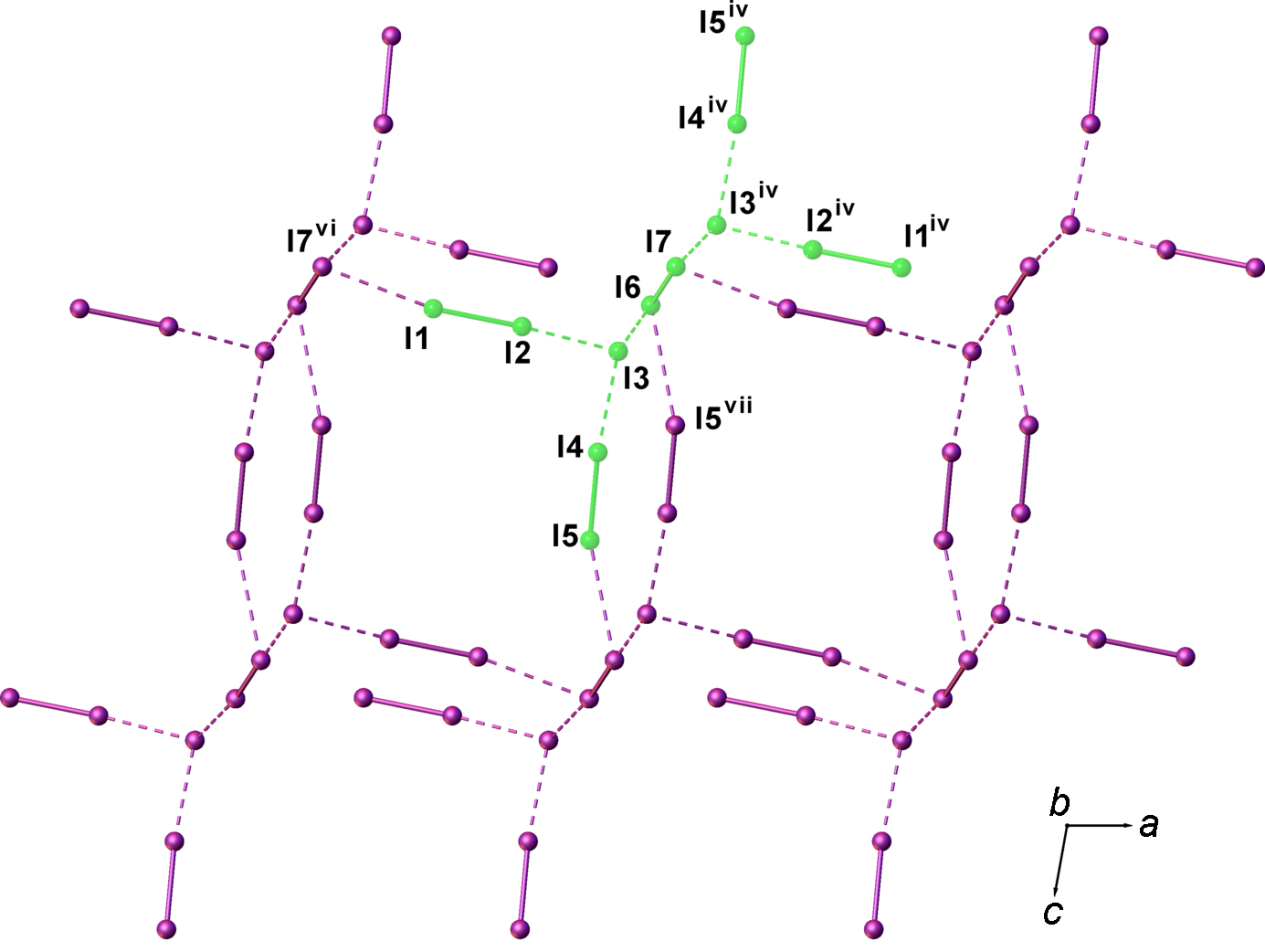
View along the *b* axis of one of the two 1D polyiodide tubes in (I) formed *via* I_12_^2−^⋯I_12_^2−^ inter­actions involving I_12_^2−^ anions of the same orientation (in this case, I_12_^2−^ is the component depicted in green as in Fig. 3 ▸). [Symmetry codes: (iv) −*x* + 1, *y* + 1, −*z* + 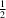; (vi) *x* − 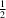, *y* − 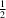, *z*; (vii) −*x* + 1, −*y* + 1, −*z* + 1.]

**Figure 5:**
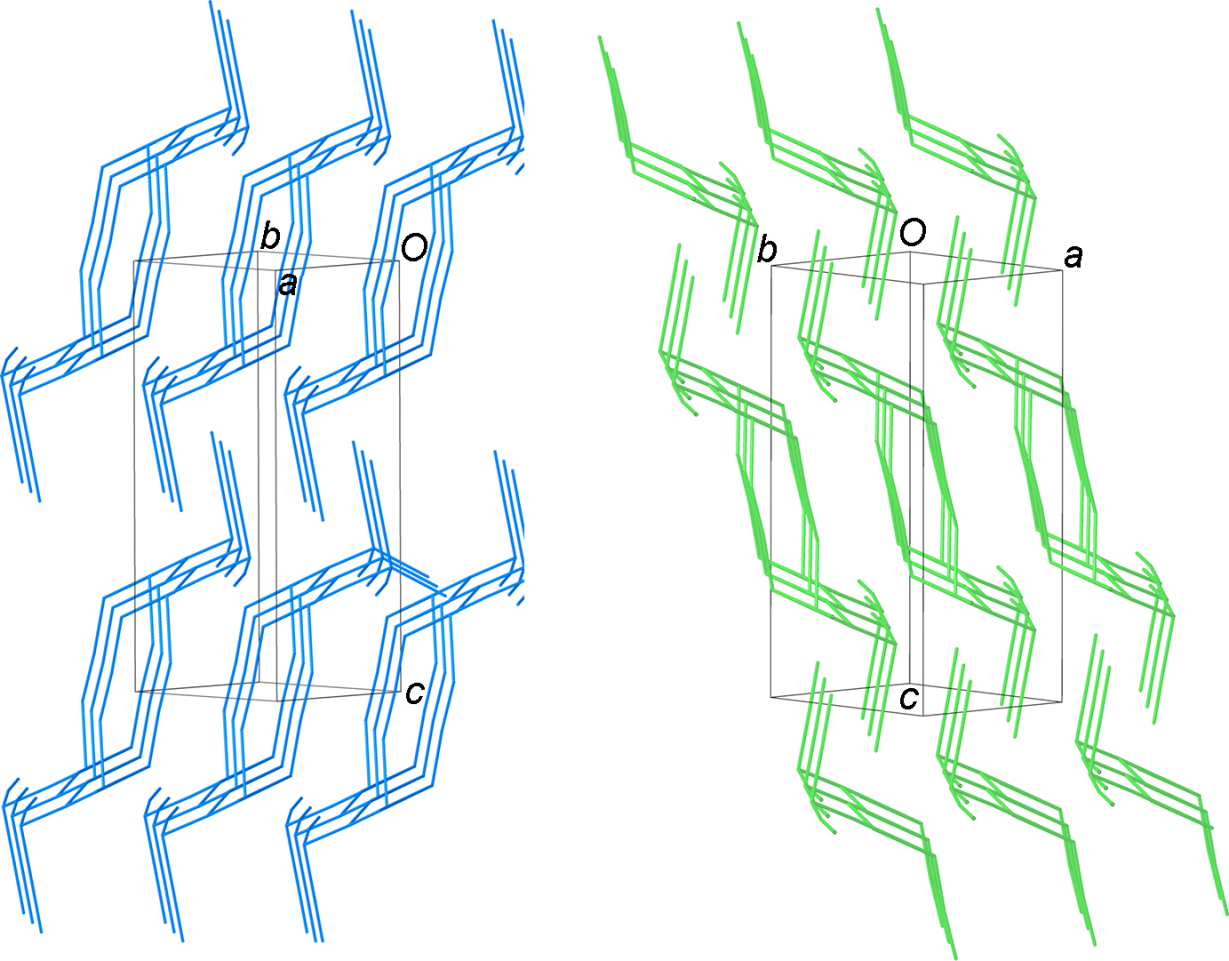
View of the two differently-oriented polyiodide 1D tubes of inter­acting I_12_^2−^ units co-existing at 50% occupancy in the crystal structure of (I). Colours are consistent with those in Fig. 3 ▸ for the differently-oriented I_12_^2−^ units generating the two 1D polyiodides tubes.

I_12_^2−^ of the same orientation (blue or green in Fig. 3 ▸) inter­act with each other *via* I⋯I inter­actions of 3.625 (2) [I1⋯I7^vi^; symmetry code: (vi) *x* − 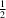, *y* − 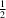, *z*] and 3.800 (2) Å [I6⋯I5^vii^; symmetry code: (vii) −*x* + 1, −*y* + 1, −*z* + 1] (Fig. 4 ▸) to give one-dimensional (1D) tubes of fused pseudo-cubic cavities defined by 8- and 14-membered polyiodide rings (Fig. 4 ▸). Two differently-oriented 1D tubes of this type therefore co-exist at 50% occupancy in the crystal structure, depending on the orientation of the generating I_12_^2−^ units; one type is approximately perpendicular and the other approximately parallel to the [110] direction (blue and green, respectively, in Fig. 5 ▸).

**Figure 6:**
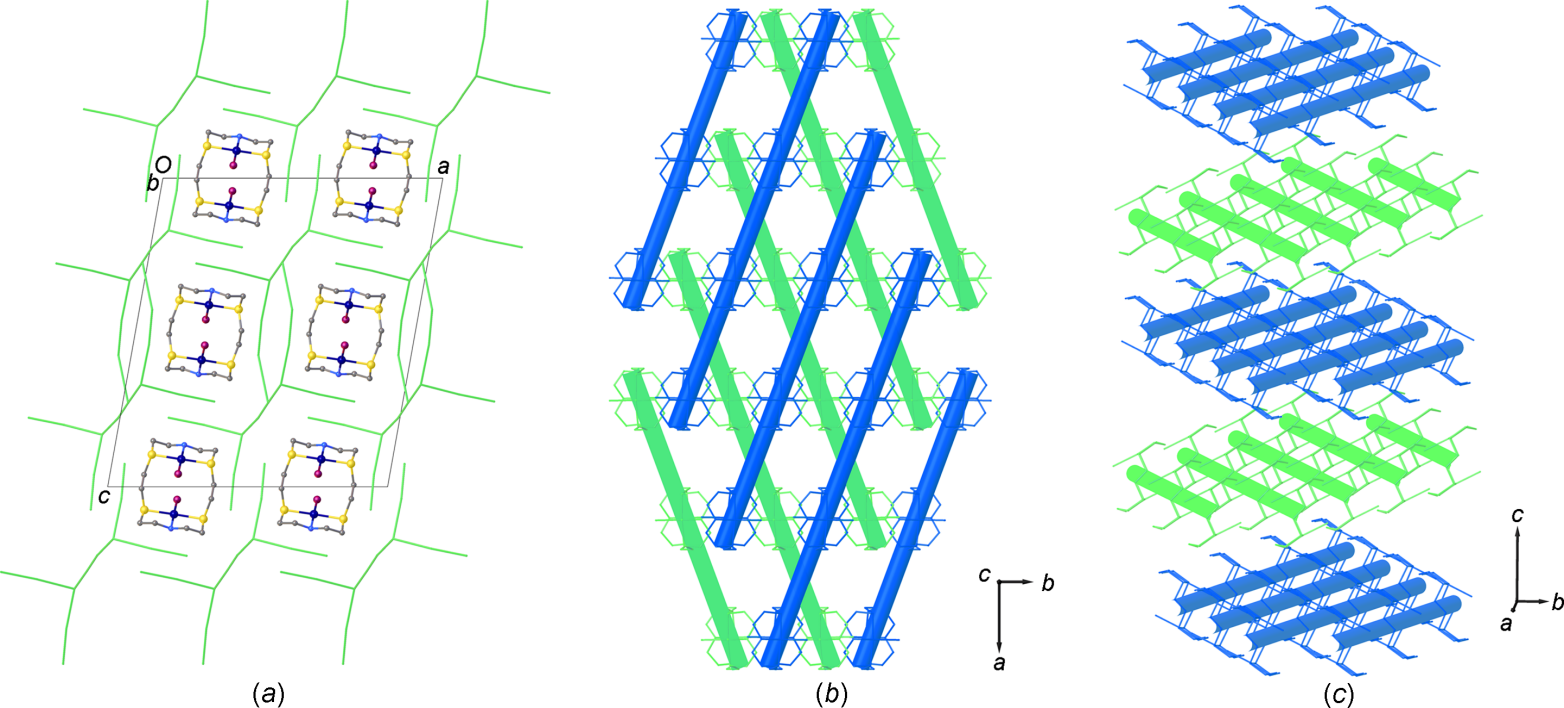
(*a*) View along the *b* axis of the crystal packing in (I), showing the relative positions between the 1D polyiodide tubes and the chains of [Pd_2_I_2_([18]aneN_2_S_4_)]^2+^ complex dications. The polyiodide network is also portrayed in parts (*b*) and (*c*) as blue and green tubes according to Fig. 3 ▸, and cations are coloured according to the type of tubes they cross.

Chains of [Pd_2_I_2_([18]aneN_2_S_4_)]^2+^ complex dications (Fig. 2 ▸) run parallel to the *b* axis crossing adjacent 1D polyiodide tubes through the pseudo-cubic cavities (Fig. 6 ▸). It is inter­esting to note that, as the I7 atom of the disordered I_2_ mol­ecule lies on a glide plane, the resulting ratio between the two components is imposed by symmetry and the maximum occupancy possible is 0.5. As a consequence, the ratio between the two types of tubes described above remains constant in the crystal structure and cannot vary between different crystals. That said, a unique crystal packing is observed in the crystal structure of (I) featuring the two sets of tubes formed by fused pseudo-cubic boxes (see above) running parallel (green) and perpendicular (blue) to the [110] direction, alternatively layered along the *c* axis [Figs. 6(*b*) and 6(*c*)].

**Figure 7:**
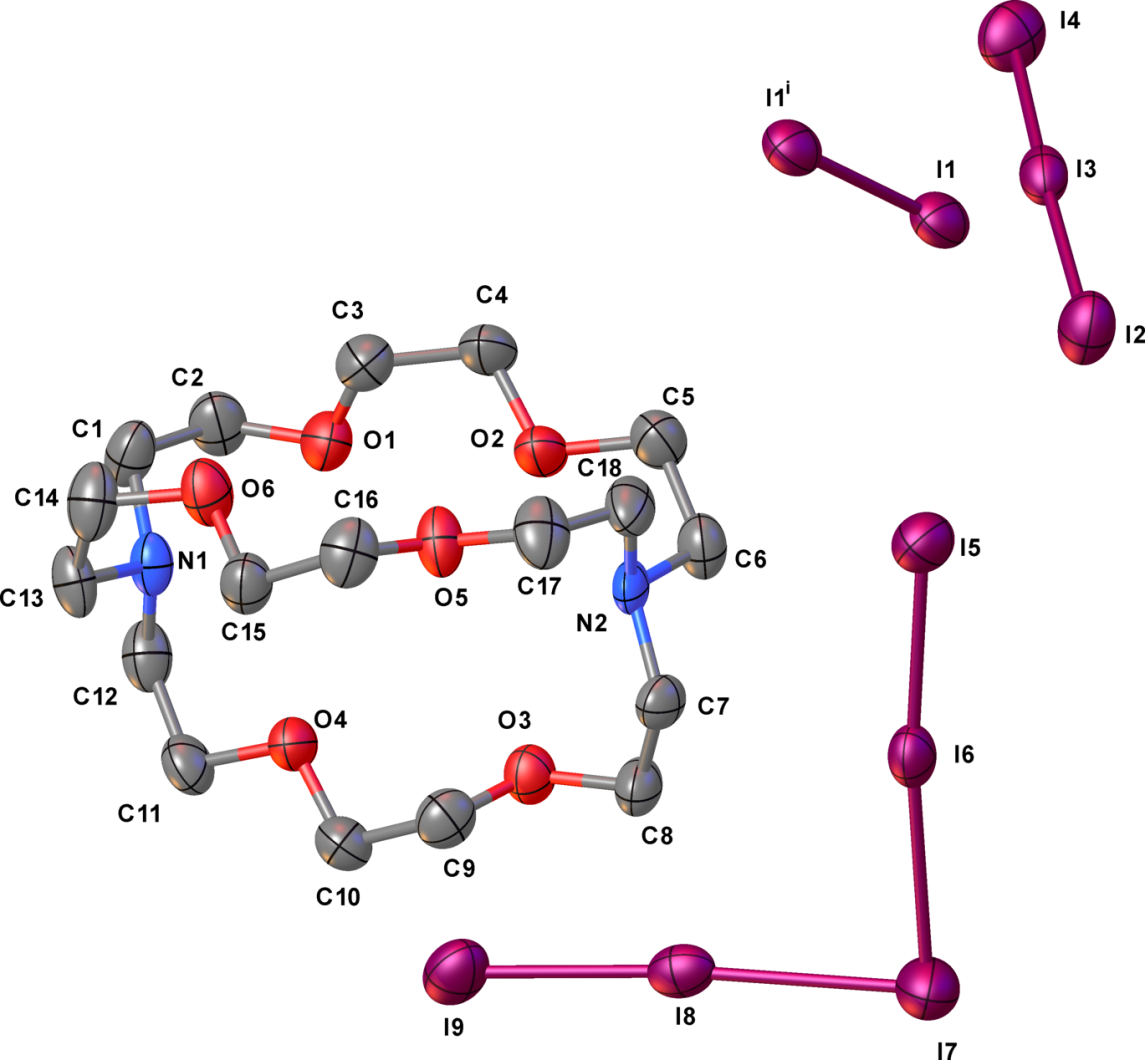
The crystal structure of (II), showing the numbering scheme adopted. Displacement ellipsoids are drawn at the 50% probability level. H atoms and the cocrystallized CH_2_Cl_2_ mol­ecules are not shown. [Symmetry code: (i) −*x* + 2, −*y* + 1, −*z*.]

To illustrate further the importance of the shape, charge and dimensions of the template cation in the polyiodide network assembly, we treated the macropolycyclic ligand [2.2.2]cryptand (Scheme 1) with I_2_ in a 1:4 molar ratio in CH_2_Cl_2_. Upon slow evaporation of the solvent at room temperature, dark prismatic crystals formed corresponding to the formulation [H_2_([2.2.2]cryptand)]I_9_·CH_2_Cl_2_, (II). An X-ray crystal structure determination (see Table 1 ▸ for crystal data) confirmed the presence of an asymmetric unit consisting of an [H_2_([2.2.2]cryptand)]^2+^ dication in which both tertiary N atoms of the starting macropolycyclic ligand are protonated (Fig. 7 ▸). Half an I_2_ mol­ecule [I1—I1^i^ = 2.7595 (11) Å; symmetry code: (i) −*x* + 2, −*y* + 1, −*z*], an asymmetric triiodide [I2—I3—I4: I2—I3 = 2.9799 (8) and I3—I4 = 2.8629 (8) Å], a ‘V-shaped’ penta­iodide consisting of an iodide anion (I7) inter­acting with two diiodine mol­ecules [(I^−^)·(I_2_)_2_] (I5—I6 and I8—I9) [I5—I6 = 2.8015 (8), I8—I9 = 2.8001 (8), I6—I7 = 3.0952 (8) and I7—I8 = 3.0940 (9) Å] and a cocrystallized CH_2_Cl_2_ solvent mol­ecule define the [H_2_([2.2.2]cryptand)](I_3_)(I)(I_2_)_2.5_·CH_2_Cl_2_ (II) stoichiometry for the obtained polyiodide salt (see Table 2 ▸ for selected geometric parameters).

**Figure 8:**
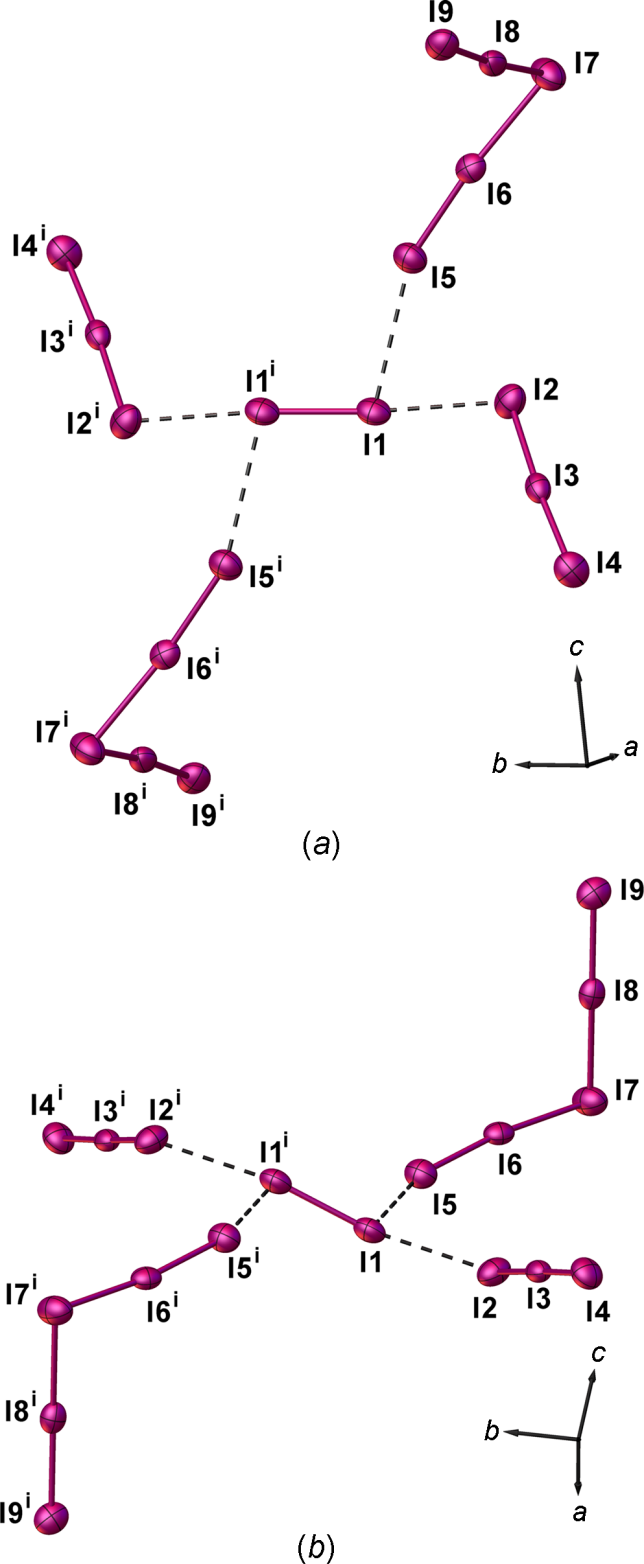
(*a*)/(*b*) Views of the I_18_^4−^ polyiodide in (II) formed by inter­action of a central diiodine mol­ecule with two I_3_^−^ and two I_5_^−^ [(I^−^)·(I_2_)_2_] species, showing the numbering scheme adopted. Displacement ellipsoids are drawn at the 50% probability level. [Symmetry code: (i) −*x* + 2, −*y* + 1, −*z*.]

In (II), all three diiodine mol­ecules are slightly elongated with respect to the I—I distance found in the crystal structure of ortho­rhom­bic I_2_ [2.715 (6) Å] (Blake *et al.*, 1998*b* ▸). Each I1 atom inter­acts with an asymmetric triiodide unit at the I2 atom to afford a ‘Z-shaped’ I_8_^2−^ dianion [I1⋯I2 = = 3.4123 (9) Å] that can be regarded as an I_3_^−^·I_2_·I_3_^−^ [(I_3_^−^)_2_·(I_2_)] complex (Savastano *et al.*, 2022 ▸). Additional longer contacts of 3.907 (1) Å, still within the sum of the van der Waals radii for iodine, between each I1 atom and the terminal iodine (I5) of a penta­iodide moiety, lead to an overall discrete ‘grasshopper-shaped’ I_18_^4−^ polyiodide. This can be envisaged as an [(I_8_^2−^)·(I_5_^−^)_2_] with a long contact between the I_8_^2−^ anion and the two I_5_^−^ moieties or, in terms of fundamental building blocks, as an [(I^−^)_2_·(I_3_^−^)_2_·(I_2_)_5_] adduct (Fig. 8 ▸). I_18_^4−^ polyiodides are quite rare in the literature: in [Co(12C4)_2_]_2_(I_18_) (12C4 is 12-crown-4), a unique central planar I_9_^−^ [(I^−^)·(I_2_)_4_] is attached to four triiodides at I⋯I distances of 3.240 (4)–3.478 (4) Å, and the [(I_9_^−^)(I_3_^−^)_4_] units are connected *via* two bridging I_3_^−^ to form polymeric chains of I_18_^4−^ = [(I_9_^−^)(I_3_^−^)_2/1_(I_3_^−^)_2/2_] (Fiolka *et al.* 2011 ▸); in [SnI_2_(mbit)_2_](I_3_)_2_·
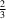
I_2_ [mbit is 1,1′-bis­(3-methyl-4-imidazoline-2-thione)methane], two I_8_^2−^ dianions of the type I_2_·I^−^·I_2_·I^−^·I_2_ [(I^−^)_2_·(I_2_)_3_] and related through an inversion centre are linked to each other at the iodide atoms by a bridging disordered I_2_ mol­ecule *via* non-negligible I⋯I inter­actions of 3.55 (1) Å (Bigoli *et al.*, 1998 ▸).

**Figure 9:**
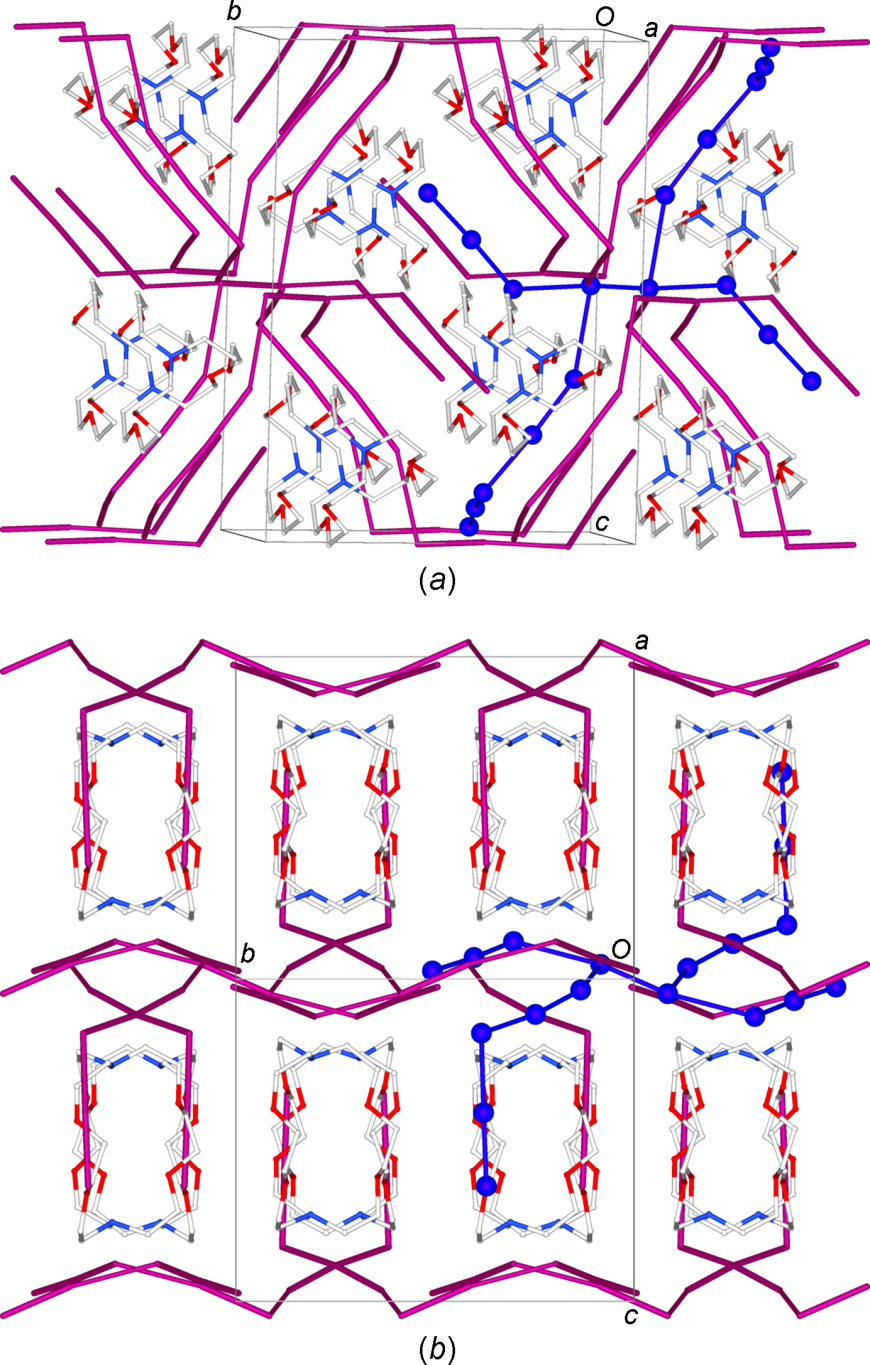
Views along approximately (*a*) the *a* axis and (*b*) the [101] direction of the crystal packing in (II). The blue colour and the ball-and-stick representation have been used for one of the discrete I_18_^4−^ polyiodide units to better highlight its atomic connectivity in the crystal packing. H atoms and the cocrystallized CH_2_Cl_2_ mol­ecules are not shown.

The discrete I_18_^4−^ polyiodide units are located side-by-side and inter­digitated along the [101] direction, with [H_2_([2.2.2]cryptand)]^2+^ dications sitting in the resulting voids (Fig. 9 ▸).

### FT–Raman spectroscopy

**Figure 10:**
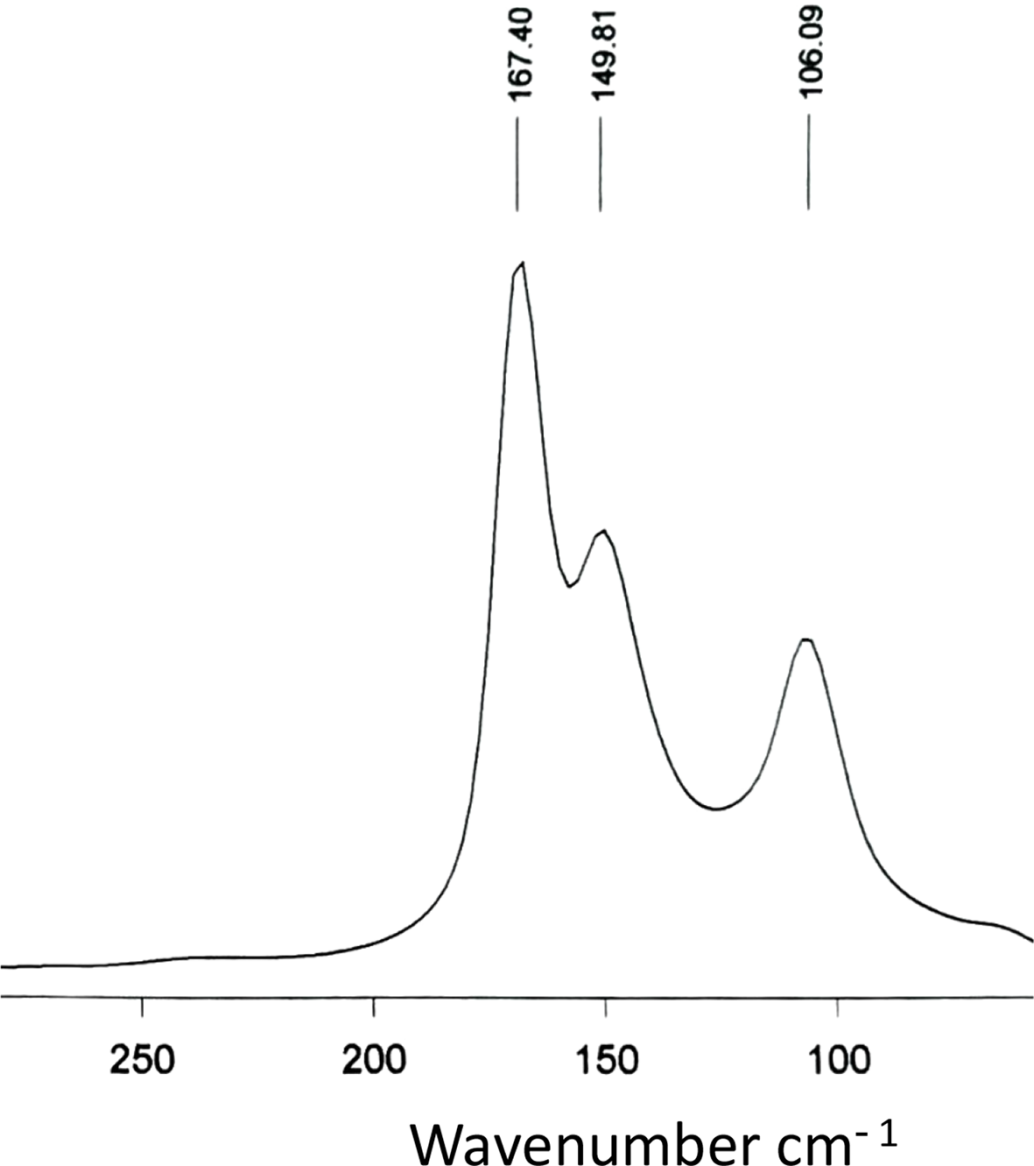
FT–Raman spectrum of (II) in the low frequency region.

Despite the high number of extended polyiodides that have been structurally characterized, and the associated crystal structure data available, the assignment of higher mol­ecular polyiodides (higher than I_3_^−^) with their own distinctive structural features is still a matter of debate (Savastano *et al.*, 2022 ▸). The reductionist approach whereby higher polyiodides are considered as aggregates of I_2_, I^−^ and I_3_^−^ held together by I⋯I inter­actions of varying strengths, from rather strong (up to *ca* 3.3–3.4 Å) (covalent inter­actions) to fairly weak (up to the van der Waals contact distance, *ca* 4 Å) (supra­molecular inter­actions), is still the most reasonable and least arbitrary. On the basis of structural data, all known higher discrete polyiodides can be regarded, therefore, as weak or medium-weak adducts of the type [(I^−^)_*n*–*y*_·(I_3_^−^)_*y*_·(I_2_)_*m*–*y*_] ≡ [I_2*m*+*n*_]^*n*−^ (*n*, *m* > 0), whose geometrical and topological features can be very different and often unpredictable (Arca *et al.* 2006 ▸). This way of considering higher polyiodides from a structural point of view is strongly supported by spectroscopic evidence. In particular, FT–Raman spectroscopy confirms that extended polyiodides do not have distinctive vibrational properties other than those of perturbed (slightly elongated) I_2_ mol­ecules and symmetric/slightly asymmetric I_3_^−^. Perturbed I_2_ mol­ecules are characterized by only one strong band in the range 180–140 cm^−1^ in the FT–Raman spectrum, the wavenumber depending on the extent of the I⋯I elongation; for linear and symmetric I_3_^−^, only the Raman-active symmetric stretch (ν_1_) occurs near 110 cm^−1^, while the anti­symmetric stretch (ν_3_) and the bending deformation (ν_2_) are only IR-active (Aragoni *et al.*, 2023*b* ▸). The latter two modes also become Raman-active for slightly asymmetric I_3_^−^ and they are found near 134 (ν_3_) and 80 cm^−1^ (ν_2_), having medium and medium–weak intensities, respectively. Highly asymmetric I_3_^−^ ions show only one band in their FT–Raman spectra in the range 180–140 cm^−1^, so that they should be regarded as weak (I^−^)·I_2_ adducts. To date, FT–Raman spectra of polyiodides of the general formula [I_2*m*+*n*_]^*n*−^ show peaks in the low wavenumber region with either one strong peak in the range 180–140 cm^−1^ or the characteristic peaks due to both perturbed I_2_ and symmetric/slightly asymmetric I_3_^−^. They would therefore be better described as [(I^−^)_*n*_·(I_2_)_*m*_] or [(I^−^)_*n*–*y*_·(I_3_^−^)_*y*_·(I_2_)_*m*–*y*_] (*n* > *y* ≠ 0)/[(I_3_^−^)_*n*_·(I_2_)_*m*–*n*_] (*n* = *y* ≠ 0) systems. The polyiodides here described are no exception. The FT–Raman spectrum of (I) features only a strong and broad peak centred at 169 cm^−1^ indicative of the presence of differently perturbed I_2_ mol­ecules (Fig. S1 in the supporting information). The FT–Raman spectrum of (II) is shown in Fig. 10 ▸. The two peaks at about 167 and 150 cm^−1^ can be assigned to the stretching vibration of the two differently elongated I_2_ mol­ecules I5—I6/I6—I7 and I1—I1^i^ [symmetry code: (i) −*x* + 2, −*y* + 1, −*z*], respectively. These data correspond closely to the established linear correlation ν(I–I)/cm^−1^*versus d*(I–I)/Å for weak or medium–weak adducts (Arca *et al.*, 2006 ▸). The peak at 106 cm^−1^ can be attributed to the symmetric stretch (ν_1_) of the I_3_^−^ ion (I2—I3—I4), thus confirming the description of the I_18_^4−^ polyiodide as an [(I^−^)_2_·(I_3_^−^)_2_·(I_2_)_5_] adduct.

## Conclusions

In this article, we confirm the structural variety of extended polyiodides that can be generated by changing the shape, charge and dimension of the cation template, as well as the synthetic strategy adopted and the experimental conditions. Although it is still often difficult to characterize [I_2*m*+*n*_]^*n*−^ polyiodides higher than I_3_^−^ on the grounds of any distinctive structural parameters, such as I—I bond distances, FT–Raman spectroscopy appears to confirm their characterization as aggregates of I_2_, I^−^ and (symmetric or slightly asymmetric) I_3_^−^ building blocks held together by I⋯I inter­actions of varying strengths. On the other hand, FT–Raman spectroscopy cannot provide any information on the topological features of extended polyiodides. The two techniques should therefore be used together in the analysis of this kind of compound.

## Supplementary Material

Crystal structure: contains datablock(s) I, II, global. DOI: 10.1107/S2053229624004194/vp3036sup1.cif

Structure factors: contains datablock(s) i. DOI: 10.1107/S2053229624004194/vp3036isup2.hkl

Structure factors: contains datablock(s) ii. DOI: 10.1107/S2053229624004194/vp3036iisup3.hkl

Additional FT-Raman spectrum. DOI: 10.1107/S2053229624004194/vp3036sup4.pdf

CCDC references: 2353843, 2353842

## Figures and Tables

**Table 1:** Experimental details For both structures: *Z* = 4. Experiments were carried out with Mo *K*α radiation.

	(I)	(II)
Crystal data		
Chemical formula	[Pd_2_I_2_(C_12_H_26_N_2_S_4_)](I)_2_·5I_2_	C_18_H_38_N_2_O_6_^2+^·I_3_^−^·I^−^·2.5I_2_·CH_2_Cl_2_
*M*_r_	2315.99	1605.53
Crystal system, space group	Monoclinic, *C*2/*c*	Monoclinic, *P*2_1_/*n*
Temperature (K)	220	293
*a*, *b*, *c* (Å)	21.609 (4), 8.198 (3), 24.151 (3)	13.831 (2), 14.820 (2), 20.266 (3)
β (°)	100.170 (13)	96.70 (1)
*V* (Å^3^)	4211.1 (18)	4125.6 (10)
μ (mm^−1^)	11.33	6.92
Crystal size (mm)	0.26 × 0.14 × 0.13	0.2 × 0.15 × 0.11

Data collection		
Diffractometer	STOE STADI4 4-circle	Bruker APEXII CCD
Absorption correction	Integration (*REDU4*; Stoe & Cie, 1996 ▸)	Empirical (using intensity measurements) (*SADABS*; Bruker, 2001 ▸)
*T*_min_, *T*_max_	0.222, 0.306	0.569, 1.000
No. of measured, independent and observed [*I* > 2σ(*I*)] reflections	4584, 3715, 3059	30347, 8100, 5518
*R*_int_	0.029	0.047
(sin θ/λ)_max_ (Å^−1^)	0.595	0.617

Refinement		
*R*[*F*^2^ > 2σ(*F*^2^)], *wR*(*F*^2^), *S*	0.051, 0.136, 1.08	0.033, 0.088, 1.00
No. of reflections	3715	8100
No. of parameters	163	349
H-atom treatment	H-atom parameters constrained	H atoms treated by a mixture of independent and constrained refinement
Δρ_max_, Δρ_min_ (e Å^−3^)	1.35, −1.62	1.37, −1.20

Computer programs: *STADI4* (Stoe & Cie, 1996 ▸), *SAINT* (Bruker, 2001 ▸), *REDU4* (Stoe & Cie, 1996 ▸), *SHELXS97* (Sheldrick, 1997 ▸), *SHELXT2018* (Sheldrick, 2015*a* ▸), *SHELXL2018* (Sheldrick, 2015*b* ▸) and *OLEX2* (Dolomanov *et al.*, 2009 ▸).

**Table 2:** Selected geometric parameters (Å, °)

**[Pd_2_I_2_([18]aneN_2_S_4_)](I)_2_·(I_2_)_5_, (I)**			
Pd—I	2.5722 (14)	S7—C8	1.816 (12)
Pd—N1	2.076 (9)	C8—C9	1.511 (16)
Pd—S4	2.314 (3)	I—I^ii^	3.545 (2)
Pd—S7^i^	2.313 (3)	I1—I2	2.7899 (15)
N1—C2	1.459 (15)	I2—I3	3.1214 (16)
N1—C9^i^	1.489 (16)	I3—I4	3.205 (2)
C2—C3	1.514 (17)	I4—I5	2.7644 (16)
C3—S4	1.809 (11)	I3—I6	3.351 (3)
S4—C5	1.838 (10)	I6—I7	2.771 (4)
C5—C6	1.507 (15)	I7—I3^iii^	3.432 (3)
C6—S7	1.796 (10)		
			
N1—Pd—S4	86.8 (3)	S7^i^—Pd—I	92.20 (8)
N1—Pd—S7^i^	87.7 (3)	S7^i^—Pd—S4	173.88 (11)
S4—Pd—I	93.57 (8)		
			
**[H_2_([2.2.2]cryptand)](I_3_)(I)(I_2_)_2.5_·CH_2_Cl_2_, (II)**			
I3—I2	2.9799 (8)	O1—C2	1.402 (8)
I3—I4	2.8629 (8)	O1—C3	1.422 (8)
I6—I5	2.8015 (8)	O2—C4	1.426 (8)
I6—I7	3.0952 (8)	O2—C5	1.430 (8)
I8—I9	2.8001 (8)	O3—C9	1.415 (8)
I8—I7	3.0940 (9)	O3—C8	1.431 (8)
I1—I1^iv^	2.7595 (11)	O4—C11	1.421 (8)
N1—C1	1.495 (9)	O4—C10	1.428 (8)
N1—C12	1.493 (8)	O5—C17	1.421 (8)
N1—C13	1.507 (8)	O5—C16	1.403 (8)
N2—C6	1.500 (8)	O6—C15	1.408 (8)
N2—C7	1.495 (8)	O6—C14	1.429 (8)
N2—C18	1.506 (8)		
			
I4—I3—I2	176.54 (2)	I9—I8—I7	175.52 (2)
I5—I6—I7	173.63 (2)	I8—I7—I6	89.70 (2)

Symmetry codes: (i) −*x* + 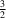, −*y* + 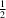, −*z* + 1; (ii) −*x* + 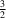, −*y* − 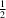, −*z* + 1; (iii) −*x* + 1, *y* + 1, −*z* + 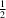; (iv) −*x* + 2, −*y* + 1, −*z*.

## Data Availability

The authors confirm that the data supporting the findings of this study are available within the article and its supplementary materials.
