# Supplementary material for: Formation of extended polyiodides at large cation templates
Source: Acta Crystallogr C Struct Chem. 2024 May 13;80(Pt 7):311–8. doi: 10.1107/S2053229624004194 (PMC11225612; doi:10.1107/S2053229624004194)
Supplement: Supplementary file 4 [file c-80-00311-sup4.pdf]

STRUCTURAL CHEMISTRY

Volume 80 (2024)

Supporting information for article:

Formation of extended polyiodides at large cation templates

Alexander J. Blake, Carlo Castellano, Vito Lippolis, Enrico Podda and Martin Schroder

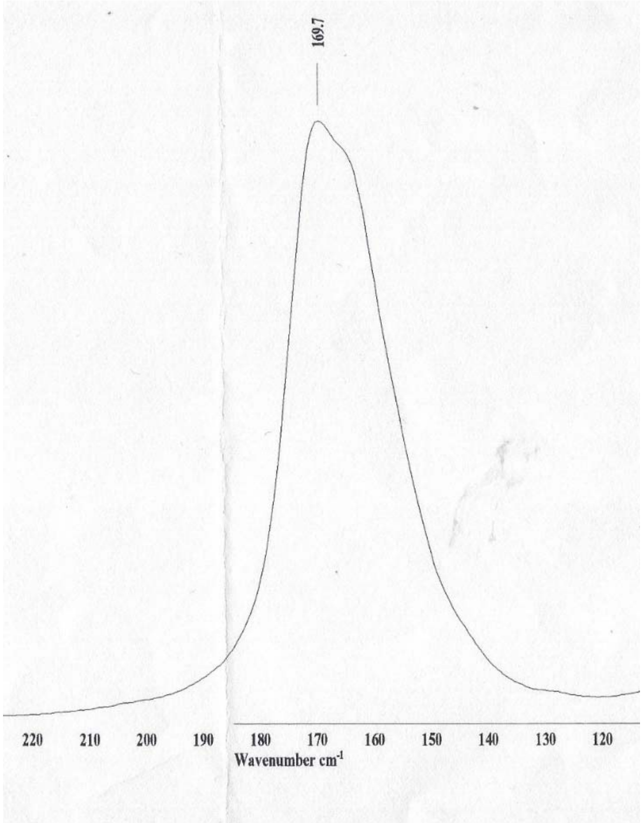

Figure S1

FT-Raman spectrum of the compound $[Pd_2I_2([18]aneN_2S_4)](I)_2{\cdot}(I_2)_5$ in the low frequency region.
